# Symptom burden, service use and care dissatisfaction among older adults with cancer, cardiovascular disease, respiratory disease, dementia and neurological disease during the last 3 months before death: A pooled analysis of mortality follow-back surveys

**DOI:** 10.1177/02692163241246049

**Published:** 2024-04-28

**Authors:** Mitsunori Miyashita, Catherine J Evans, Deokee Yi, Barbara Gomes, Wei Gao

**Affiliations:** 1 Cicely Saunders Institute of Palliative Care, Policy and Rehabilitation, Florence Nightingale Faculty of Nursing, Midwifery & Palliative Care, King’s College London, London, UK; 2 Department of Palliative Nursing, Health Sciences, Tohoku University Graduate School of Medicine, Sendai, Miyagi, Japan, Japan; 3 Faculty of Medicine, University of Coimbra, Coimbra, Portugal; 4 School of Public Health, Jiangxi Medical College, Nanchang University, Nanchang, China

**Keywords:** Palliative care, neoplasms, non-malignant diseases, bereavement, symptoms, satisfaction, survey

## Abstract

**Background:**

Variation in the provision of care and outcomes in the last months of life by cancer and non-cancer conditions is poorly understood.

**Aims:**

(1) To describe patient conditions, symptom burden, practical problems, service use and dissatisfaction with end-of-life care for older adults based on the cause of death. (2) To explore factors related to these variables focussing on the causes of death.

**Design:**

Secondary analysis of pooled data using cross-sectional mortality follow-back surveys from three studies: QUALYCARE; OPTCare Elderly; and International Access, Right, and Empowerment 1.

**Setting/participants:**

Data reported by bereaved relatives of people aged ⩾75 years who died of cancer, cardiovascular disease, respiratory disease, dementia or neurological disease.

**Results:**

The pooled dataset contained 885 responses. Overall, service use and circumstances surrounding death differed significantly across causes of death. Bereaved relatives reported symptom severity from moderate to overwhelming in over 30% of cases for all causes of death. Across all causes of death, 28%–38% of bereaved relatives reported some level of dissatisfaction with care. Patients with cardiovascular disease and dementia experienced lower symptom burden and dissatisfaction than those with cancer. The absence of a reliable key health professional was consistently associated with higher symptom burden (*p* = 0.002), practical problems (*p* = 0.001) and dissatisfaction with care (*p* = 0.001).

**Conclusions:**

We showed different trajectories towards death depending on cause. Improving symptom burden and satisfaction in patients at the end-of-life is challenging, and the presence of a reliable key health professional may be helpful.

**What is already known about the topic?**

- Although there have been many mortalities follow-back surveys of patients with cancer, few comparative analyses with non-cancer patients have been conducted.

**What this paper adds?**

- This study demonstrates that service use and circumstances surrounding death differed significantly across different causes of death.
- After adjustment, patients with cardiovascular disease and dementia experienced lower symptom burden and dissatisfaction than those with cancer.
- The absence of a reliable key health professional was consistently associated with higher symptom burden, practical problems and dissatisfaction with care.

**Implications for practice, theory or policy**

- Patient trajectories at the end-of-life differ across diseases. Palliative care should be tailored and adapted to the course of these trajectories.
- The presence of a key health professional might reduce symptom burden and practical problems and improve dissatisfaction with care across diseases.

## Background

Rapid population ageing is a global phenomenon. The highest projected rise in serious health related suffering is among people aged 70 years (183% increase between 2016 and 2026).^1^ Ageing and the consequently rising mortality rates have increased demand for palliative care, both for cancer- and non-cancer diseases.^2,3^

The World Health Organization proposed that palliative care is an essential component of comprehensive health services for non-communicable diseases, with an emphasis on primary healthcare and community- or home-based care.^4^ With population ageing, non-cancer conditions are projected to have the greatest proportional rise in serious health related suffering, with dementia the highest (six million people, 264% increase between 2016 and 2060).^1^ However, the provision of palliative care for patients with non-cancer conditions remains insufficient, globally representing 69% of people in need of palliative care.^5^ Furthermore, given the different trajectories of diseases^6,7^ and changing palliative care practices, national end-of-life care strategies require understanding on symptom distress and system based solutions to address inequities in providing palliative care. Consequently, it is vital to understand comprehensively the experiences and quality of care among patients with cancer and non-cancer diseases near end-of-life. This understanding should be evaluated within the context of each healthcare system, considering the implications of rapid population ageing. These considerations are crucial to design effective end-of-life care policies and optimise service provision, especially in settings with limited resources.

Donabedian proposed a framework for evaluating care quality, which comprises structure, process and outcome of care.^8^ Palliative care includes the places of care delivery for general and specialised services and the provision of multidisciplinary care. The outcome of care encompasses symptom burden, quality of life and patients’ and families’ satisfaction with care.^9,10^ However, measuring the process and outcomes of palliative care at the end-of-life using existing databases or prospective data collection is often challenging because of the vulnerability of these patients and the complexity of the palliative care delivery system. Therefore, retrospective approaches, such as mortality follow-back surveys, are commonly used.^11^

Most mortality follow-back surveys focus on cancer or a single non-cancer disease, with few including several non-cancer diseases.^12,13^ Additionally, these surveys consider both younger and older adults. Furthermore, even when several non-cancer diseases are included, analyses are limited to comparisons between patients with and without cancer, owing to small sample sizes.^14–16^ Diff-erences between causes of death have not been examined to the best of our knowledge. Therefore, in this secondary analysis, we pooled data from three studies using mortality follow-back surveys^17–20^

Our aims were to: (1) gain a thorough understanding of service use, patient circumstances, symptom burden, practical problems and dissatisfaction with end-of-life care for older adults by cause of death and (2) explore factors related to symptom burden, practical problems and dissatisfaction, focussing on the causes of death.

## Methods

### Study design

We conducted a secondary analysis of pooled data using cross-sectional mortality follow-back surveys from three studies: QUALYCARE^17^; OPTCare Elderly^18^; and the Inter-national Access, Right and Empowerment 1 (IARE 1).^19,20^

### Settings

The three studies focussed on deaths in community settings. In the QUALYCare and OPTCare elderly studies stratifying the sampling to include all deaths at home, in-patient hospice and care homes. And in QUALYCare a random sample of deaths in acute hospital. IARE1 included all places of death following discharge from acute hospital. The QUALYCARE and IARE 1, studies were conducted in London, UK providing diverse ethnic and socio-economic samples, while the OPTCare Elderly study was conducted in two diverse geographical areas in Southern England encompassing rural/urban and city.

### Study population and sampling

We analysed data from bereaved relatives of individuals aged ⩾75 years whose cause of death, as identified from their death certificate, was cancer (C00–C97), cardiovascular disease (I00–I99), respiratory disease (J00–J99), dementia (F00–F03) or neurological disease (G00–G99).

The participants of the QUALYCARE survey were bereaved relatives of individuals aged ⩾18 years who died of cancer within 1 year from March 2009 to March 2010. Postal questionnaires were sent between January and July 2010, 4–10 months after the death occurred. We extracted and analysed data from those whose deceased relatives had been ⩾75 years. Four primary care trusts were selected specifically for their differing rates of cancer home deaths and levels of deprivation within London. Subsequently, the sample was stratified by primary care trust and place of death.

The participants of the OPTCare Elderly study were bereaved relatives of individuals aged ⩾75 years who died of cancer or other causes. Postal questionnaires were sent in October 2012, 4–10 months after the death occurred. The study sites included both urban and rural areas, which allowed for a comparison between different geographical areas. The Office for National Statistics randomly selected bereaved relatives of individuals who died at the designated study sites within 6-months. Additionally, death registration data were used to identify the sampling frame.

The participants of the IARE 1 study were bereaved relatives of individuals aged ⩾65 years who died of cancer or other causes and had accessed specialist palliative care services before their death. The study recruited patients who received palliative care at two hospitals in London from 2012 to 2013. We extracted and analysed data from those whose deceased relatives had been ⩾75 years. A mortality follow-back survey was conducted 4–10 months after the death occurred.

Other information about the study population and sampling procedure is described in Supplemental Table 1. More detailed information about the three original studies is provided elsewhere.^17,19–21^

### Approval and ethical considerations

All studies were approved by the National Health Sys-tem Research Ethics Committee, London, Dulwich (ref: QUALYCARE 09/H0808/85, OPTCare Elderly 12/LO/1367, IARE 1 12/LO/0044) and King’s College London.

### Measurements

The underlying causes of death were identified based on the Office for National Statistics registry for the QUALYCARE and OPTCare Elderly studies and based on hospital records for the IARE 1 study.

The primary outcomes were symptoms and practical problems in the last week, and dissatisfaction with care. However, because the aim of this study was a thorough understanding of the circumstances of patients near death, we examined the following variables using a modified version of a questionnaire developed by Ann Cartwright, widely used to measure patients’ experiences in their last year of life from the perspective of bereaved family members.^22–25^

#### Symptoms and practical problems in the last week before death

The Palliative Outcome Scale (POS)^26^ and POS for symptoms (POS-S)^27^ were used to measure patient symptom burden and practical problems experienced in the last week before death. The POS and POS-S comprise 10 and 8 items, respectively. Bereaved relatives rated the items from 0 to 4, considering time-wasted appointments (0–3) and practical matters (0–3) from their perspective. Additionally, the 18 items were classified into two components: symptoms and practical problems.^28^

#### Dissatisfaction with care

The respondents were asked whether (Yes or No) there were any aspects of care they felt unhappy with during the last 3 months of their relative’s life.

#### Service use in the last 3 months

The Client Receipt Service Inventory was used to obtain information on healthcare services received by individuals in their last 3 months of life.^29,30^ Hospice and palliative care teams, Macmillan nurses, Marie Curie nurses and other specialist nurses were considered as specialised palliative care services. Respondents were asked about the presence of a key professional: ‘Do you feel he/she had a key contact person (health professional) he/she could rely on to get things done?’

#### Circumstances surrounding death

Information was ob-tained on death-related circumstances, such as place of death, patient’s awareness of their prognosis in the last week and unconsciousness or coma in the last week.

#### Participant characteristics

The collected data included the patient’s gender, age, religion, ethnicity, marital status, financial status and length of illness and the bereaved relative’s gender, age, relationship, religion and ethnicity.

### Analysis

First, we analysed data on the participants’ characteristics, service use in the last 3 months and death-related circumstances. Using Fisher’s exact test, we compared the data by cause of death. Regarding symptoms and practical problems, we provided summary statistics of each item, as well as subtotal and total scores. We compared these scores with the causes of death using an analysis of variance. Additionally, we calculated the percentage of items with scores ⩾2 from 0 to 4, indicating a moderate, severe or overwhelming problem for each POS and POS-S item.

The factors related to symptoms, practical problems and dissatisfaction with care were explored using bivariate and multivariate analyses to identify independently associated variables. To assess symptoms and practical problems, we set the subtotal scores as dependent variables, performed a t-test or analysis of variance for bivariate analyses and conducted multiple linear regression analyses. Regarding dissatisfaction, we set dissatisfaction (i.e. unhappy experience) as the dependent variable, used Fisher’s exact test for the bivariate analyses of cases without symptoms and practical problems and conducted multiple logistic regression analyses. The model selection strategy for all multiple regression analyses was as follows: (1) bivariate analyses assessed the presence or absence of a statistical association between the causes of death and all other variables; (2) after checking any statistical interactions to determine that the association did not differ by cause of death, the causes of death and all associated variables in the bivariate analyses were included in the first model; (3) statistical variable selection was performed using a backward procedure, with the compulsory inclusion of causes of death; and (4) sensitivity analyses were conducted according to processing method of missing values. All analyses were two-sided; statistical significance and threshold for variable selection were set at *p* < 0.05. All analyses were performed using SAS version 9.4 (SAS Institute, Cary, NC, USA).

## Results

The pooled datasets contained 885 responses from the three studies (QUALYCARE, *n* = 328; OPTCare Elderly, *n* = 406 and IARE 1, *n* = 151). The causes of death were cancer (*n* = 516), cardiovascular disease (*n* = 172), respiratory disease (*n* = 104), dementia (*n* = 51) and neurological disease (*n* = 42). The study details and causes of death are presented in Supplemental Table 1.

### Participant characteristics and circumstances surrounding death

Table 1 presents the participants’ characteristics and circumstances surrounding death. Almost all variables except for religion, ethnicity and the bereaved relative’s gender significantly differed across the causes of death.

**Table 1.** Participant characteristics (*N* = 885).

	Cancer		Cardiovascular disease		Respiratory disease		Dementia		Neurological disease		*p* value
	*n* = 516		*n* = 172		*n* = 104		*n* = 51		*n* = 42		
	*n*	%	*n*	%	*n*	%	*n*	%	*n*	%	
Patients’ characteristics											
Age at death (years)											
75–79	127	24.6	26	15.1	12	11.5	3	5.9	3	7.1	0.001
80–84	179	34.7	37	21.5	28	26.9	6	11.8	13	31.0	
85–89	127	24.6	50	29.1	24	23.1	15	29.4	12	28.6	
90–94	73	14.2	38	22.1	26	25.0	19	37.3	9	21.4	
⩾95	10	1.9	21	12.2	14	13.5	8	15.7	5	11.9	
Gender											
Man	257	49.8	74	43.0	42	40.4	13	25.5	12	28.6	0.001
Woman	259	50.2	98	57.0	62	59.6	38	74.5	30	71.4	
Marital status											
Married/with partner	191	37.0	37	21.5	23	22.1	12	23.5	7	16.7	0.001
Widowed	223	43.2	95	55.2	51	49.0	30	58.8	23	54.8	
Divorced/separated/never married	60	11.6	15	8.7	16	15.4	5	9.8	6	14.3	
Do not know/missing information	42	8.1	25	14.5	14	13.5	4	7.8	6	14.3	
Religion											
No religion	67	13.0	18	10.5	10	9.6	2	3.9	4	9.5	0.35
Christian	422	81.8	141	82.0	90	86.5	47	92.2	33	78.6	
Other	15	2.9	7	4.1	3	2.9	2	3.9	4	9.5	
Missing	12	2.3	6	3.5	1	1.0	0	0.0	1	2.4	
Ethnicity											
White British	454	88.0	153	89.0	93	89.4	49	96.1	34	81.0	0.21
Other ethnic groups	50	9.7	11	6.4	9	8.7	2	3.9	7	16.7	
Missing information	12	2.3	8	4.7	2	1.9	0	0.0	1	2.4	
Living circumstances											
Living alone	242	46.9	86	50.0	38	36.5	21	41.2	9	21.4	0.001
With relatives	252	48.8	65	37.8	41	39.4	17	33.3	19	45.2	
Care home	16	3.1	19	11.1	24	23.1	12	23.5	14	33.3	
Missing information	6	1.2	2	1.2	1	1.0	1	2.0	0	0.0	
Financial status											
Living comfortably	211	40.9	88	51.2	47	45.2	20	39.2	24	57.1	0.026
Doing alright	191	37.0	57	33.1	35	33.7	18	35.3	7	16.7	
Just about getting by	57	11.1	12	7.0	9	8.7	9	17.7	5	11.9	
Finding it quite difficult/very difficult	47	9.1	7	4.1	11	10.6	1	2.0	4	9.5	
Missing information	10	1.9	8	4.7	2	1.9	3	5.9	2	4.8	
Duration of illness											
˂1 week	5	1.0	12	7.0	13	12.5	2	3.9	2	4.8	0.001
˂1 month	24	4.7	34	19.8	23	22.1	5	9.8	4	9.5	
˂6 months	156	30.2	38	22.1	18	17.3	5	9.8	2	4.8	
˂1 year	89	17.3	15	8.7	9	8.7	5	9.8	5	11.9	
˂3 years	135	26.2	34	19.8	12	11.5	9	17.7	7	16.7	
⩾3 years	92	17.8	25	14.5	25	24.0	25	49.0	22	52.4	
Missing information	15	2.9	14	8.1	4	3.9	0	0.0	0	0.0	
Awareness of prognosis											
Yes	386	74.8	67	39.0	55	52.9	13	25.5	24	57.1	0.001
No	130	25.2	105	61.1	49	47.1	38	74.5	18	42.9	
Confusion in the last week											
Always	26	5.0	13	7.6	11	10.6	27	52.9	18	42.9	0.001
Most of the time	77	14.9	33	19.2	21	20.2	17	33.3	9	21.4	
Sometimes	116	22.5	28	16.3	15	14.4	4	7.8	6	14.3	
Occasionally	139	26.9	38	22.1	33	31.7	2	3.9	3	7.1	
No, not at all	148	28.7	51	29.7	24	23.1	0	0.0	5	11.9	
Missing information	10	1.9	9	5.2	0	0.0	1	2.0	1	2.4	
Unconscious/coma in last week											
Always	6	1.2	7	4.1	1	1.0	1	2.0	3	7.1	0.007
Most of the time	66	12.8	18	10.5	9	8.7	9	17.7	9	21.4	
Sometimes	87	16.9	17	9.9	17	16.4	13	25.5	6	14.3	
Occasionally	85	16.5	19	11.1	12	11.5	6	11.8	5	11.9	
No, not at all	252	48.8	108	62.8	63	60.6	21	41.2	18	42.9	
Missing information	20	3.9	3	1.7	2	1.9	1	2.0	1	2.4	
Place of death											
Home	153	29.7	48	27.9	17	16.4	8	15.7	14	33.3	0.001
Hospice	164	31.8	87	50.6	61	58.7	10	19.6	15	35.7	
Hospital	60	11.6	34	19.8	24	23.1	33	64.7	13	31.0	
Care home	139	26.9	3	1.7	2	1.9	0	0.0	0	0.0	
Bereaved relatives’ characteristics											
Gender											
Man	158	30.6	61	35.5	37	35.6	20	39.2	10	23.8	0.67
Woman	356	69.0	111	64.5	67	64.4	31	60.8	32	76.2	
Missing information	2	0.4	0	0.0	0	0.0	0	0.0	0	0.0	
Age (years)											
⩽44	20	3.9	12	7.0	2	1.9	0	0.0	1	2.4	0.005
45–54	136	26.4	41	23.8	14	13.5	10	19.6	6	14.3	
55–64	163	31.6	54	31.4	41	39.4	17	33.3	16	38.1	
65–74	88	17.1	40	23.3	31	29.8	20	39.2	13	31.0	
75–84	86	16.7	18	10.5	12	11.5	3	5.9	6	14.3	
⩾85	19	3.7	6	3.5	4	3.9	1	2.0	0	0.0	
Missing information	4	0.8	1	0.6	0	0.0	0	0.0	0	0.0	
Relationship with the patient											
Spouse/partner	142	27.5	27	15.7	17	16.4	5	9.8	5	11.9	0.001
Son/daughter	289	56.0	124	72.1	67	64.4	35	68.6	34	81.0	
Other	85	16.5	21	12.2	20	19.2	11	21.6	3	7.1	
Religion											
No religion	89	17.3	31	18.0	11	10.6	13	25.5	7	16.7	0.29
Christian	392	76.0	128	74.4	82	78.9	37	72.6	30	71.4	
Other	23	4.5	9	5.2	7	6.7	1	2.0	5	11.9	
Missing information	12	2.3	4	2.3	4	3.9	0	0.0	0	0.0	
Ethnicity											
White British	465	90.1	156	90.7	95	91.4	50	98.0	36	85.7	0.08
Other	41	8.0	8	4.7	6	5.8	1	2.0	6	14.3	
Missing information	10	1.9	8	4.7	3	2.9	0	0.0	0	0.0	
Awareness of prognosis											
Yes	433	83.9	97	56.4	66	63.5	38	74.5	34	81.0	0.001
No	83	16.1	75	43.6	38	36.5	13	25.5	8	19.1	
Presence at the time of death											
Yes	312	60.5	85	49.4	55	52.9	18	35.3	27	64.3	0.001
No/do not know/missing information	204	39.5	87	50.6	49	47.1	33	64.7	15	35.7	

### Symptom burden, practical problems in the last week of life and dissatisfaction

Table 2 shows the symptom burden and practical problems in the last week. Regarding symptom burden, bereaved relatives reported a ⩾30% experience of symptoms with a score ⩾2 for most symptoms. However, symptom severity did not significantly differ across causes of death except for weakness, shortness of breath, nausea and vomiting.

**Table 2.** Symptom burden, practical problems and dissatisfaction.

Symptom burden	Cancer			Cardiovascular disease			Respiratory disease			Dementia			Neurological disease			*P* value
	Mean	SD	% (#)	Mean	SD	% (#)	Mean	SD	% (#)	Mean	SD	% (#)	Mean	SD	% (#)	
	*n* =	304		*n* =	84		*n* =	44		*n* =	19		*n* =	16		
Weakness	3.2	0.9	95	2.7	1.1	88	3.1	0.8	98	2.8	0.9	89	3.4	0.8	100	0.001
Drowsiness	2.5	1.2	81	2.2	1.3	71	2.5	1.1	84	2.5	1.0	89	3.0	1.3	88	0.15
Shortness breath	1.9	1.3	60	2.2	1.3	70	2.5	1.2	86	1.6	1.2	58	1.9	1.5	63	0.003
Constipation	1.5	1.4	49	1.2	1.2	40	1.3	1.3	48	0.9	1.0	21	1.5	1.5	56	0.27
Symptoms other than pain	1.4	1.2	45	1.2	1.2	36	1.5	1.2	50	1.2	0.9	26	1.9	1.1	69	0.19
Pain	1.2	1.2	43	1.0	1.1	37	1.2	1.1	39	1.1	0.7	32	0.9	1.1	31	0.31
Nausea	1.2	1.2	39	0.9	1.1	29	0.8	1.0	25	0.6	0.8	16	0.8	1.1	38	0.008
Diarrhoea	0.9	1.3	26	0.7	1.1	19	0.8	1.2	27	0.6	0.8	16	0.8	1.5	25	0.63
Vomiting	0.8	1.1	24	0.4	0.9	12	0.5	0.8	11	0.4	0.7	11	0.6	1.0	25	0.005
Feeling good as a person	1.9	1.5	51	2.0	1.4	60	2.0	1.5	55	2.4	1.5	53	2.7	1.5	38	0.09
Depression	1.5	1.3	45	1.3	1.3	39	1.2	1.2	34	1.1	1.3	37	1.4	1.5	50	0.40
Anxiety	1.4	1.2	42	1.3	1.2	38	1.2	1.2	34	1.2	1.1	37	1.7	1.6	50	0.70
Practical problems in the last week																
Family anxiety	2.7	1.3	77	2.2	1.5	60	3.5	1.2	75	3.3	1.3	68	4.4	1.0	88	0.02
Sharing feeling	1.7	1.5	51	1.9	1.6	61	2.6	1.5	50	4.2	1.1	89	4.1	1.3	81	0.001
Financial or personal matter	1.1	1.3	35	1.7	1.3	56	2.4	1.3	48	2.2	1.4	42	2.2	1.4	38	0.002
Difficulty with communication	1.8	1.4	60	1.5	1.4	49	2.0	1.4	68	2.6	1.2	84	3.0	1.4	88	0.001
Information	0.8	1.2	29	1.2	1.6	35	2.2	1.4	43	1.7	1.3	21	1.9	1.5	25	0.07
Time wasted	0.2	0.5	4	0.1	0.5	6	1.2	0.5	7	1.1	0.2	0	1.0	0.0	0	0.37
*Total score of symptom burden*	19.3	7.7		17.1	7.8		18.7	7.6		16.3	5.8		20.7	9.6		0.284
*Total score of practical problems*	8.3	3.9		8.7	3.8		8.8	3.9		10.1	2.7		11.6	4.0		0.0032
*Dissatisfaction in the 3 months*	*n* = 516			*n* = 172			*n* = 104			*n* = 51			*n* = 42			
Yes	196	38.0		51	29.7		33	31.7		14	27.5		17	40.5		0.18
No	302	58.5		112	65.1		62	59.6		35	68.6		23	54.8		
Do not know/missing information	18	3.5		9	5.2		9	8.7		2	3.9		2	4.8		
Analysed using POS complete data																
# Percentage of POS score ⩾2																

Overall, 60%–88% of bereaved relatives reported family anxiety (score: ⩾2), with family anxiety, sharing feelings, practical matters and difficulty in communication significantly differing across causes of death. Additionally, the total score for practical problems was different across causes of death (*p* = 0.003), but not the total score for symptom burden (*p* = 0.28).

Across causes of death, 28%–38% of bereaved relatives reported some dissatisfaction with care (*p* = 0.18).

### Service use in the last 3 months of life

Service use in the last 3 months of life is shown in Table 3. Across all causes of death, most patients had consulted with a general practitioner face-to-face. Other commonly used services such as hospital units, district/community nurses and ambulance services had a utilisation rate of >50%, except in dementia cases. Multidisciplinary care services such as spiritual care, occupational therapists and social workers were used by <20% of patients across all causes of death. Although 92% and 67% of individuals who died of cancer and neurological disease, respectively, used any one of the specialised care services, the proportion was lower (*p* = 0.001) for those who died of cardiovascular disease (33%), respiratory disease (33%) and dementia (10%). Across causes of death, 43%–60% of bereaved relatives reported access to reliable key health professionals (*p* = 0.02). Furthermore, the types of key health professionals based on multiple answers included general practitioners (26%), nurses (19%), non-general practitioner physicians (5%) and other professionals (14%; data not shown).

**Table 3.** Service use in the last 3 months before death.

	Cancer		Cardiovascular disease		Respiratory disease		Dementia		Neurological disease		*p* Value
	*n* =	516	*n* =	172	*n* =	104	*n* =	51	*n* =	42	
	n	%	n	%	n	%	n	%	n	%	
General service											
Face-to-face consultation with the general practitioner	461	89.3	143	83.1	94	90.4	47	92.2	40	95.2	0.13
Hospital stay	358	69.4	110	64.0	68	65.4	18	35.3	25	59.5	0.001
District nurse contact	344	66.7	73	42.4	62	59.6	20	39.2	26	61.9	0.001
Ambulance service use	312	60.5	111	64.5	67	64.4	19	37.3	26	61.9	0.044
Telephone consultation with the general practitioner	296	57.4	89	51.7	42	40.4	14	27.5	20	47.6	0.001
Outpatient clinic visit	140	27.1	39	22.7	23	22.1	5	9.8	6	14.3	0.08
Nursing home stay	69	13.4	41	23.8	27	26.0	30	58.8	14	33.3	0.001
Other nurse contacts	63	12.2	30	17.4	21	20.2	13	25.5	11	26.2	0.001
Intensive care unit stay	61	11.8	22	12.8	14	13.5	3	5.9	2	4.8	0.001
Day care visit	45	8.7	0	0.0	1	1.0	0	0.0	1	2.4	0.001
Residential home stay	22	4.3	14	8.1	15	14.4	15	29.4	11	26.2	0.001
Multidisciplinary care											
Consultation for spiritual care	100	19.4	22	12.8	12	11.5	10	19.6	10	23.8	0.06
Consultation with the occupational therapist	87	16.9	20	11.6	13	12.5	6	11.8	10	23.8	0.11
Consultation with the social worker	84	16.3	22	12.8	19	18.3	12	23.5	9	21.4	0.14
Consultation with the physiotherapist	59	11.4	18	10.5	14	13.5	1	2.0	8	19.1	0.07
Consultation with the other professionals	51	9.9	13	7.6	6	5.8	8	15.7	5	11.9	0.06
Consultation with the psychologist or counsellor	16	3.1	0	0.0	1	1.0	0	0.0	2	4.8	0.044
Consultation with the psychiatrist	11	2.1	2	1.2	4	3.9	2	3.9	7	16.7	0.001
Specialist palliative care											
Consultation with the palliative care team	237	45.9	9	5.2	10	9.6	3	5.9	13	31.0	0.001
Consultation with Macmillan or other specialist nurses	208	40.3	12	7.0	12	11.5	1	2.0	3	7.1	0.001
Hospice stay	153	29.7	3	1.7	4	3.9	1	2.0	2	4.8	0.001
Consultation with Marie Curie	78	15.1	3	1.7	2	1.9	1	2.0	3	7.1	0.001
Consultation with the any other specialist palliative care providers	475	92.1	57	33.1	34	32.7	5	9.8	28	66.7	0.001
Relationship with professional service											
Presence of a key health professional	287	55.6	74	43.0	45	43.3	29	56.9	25	59.5	0.02
Discussion of death between professional and patient	261	50.6	31	18.0	23	22.1	0	0.0	5	11.9	0.001
Discussion of death between professional and relative	384	74.4	93	54.1	55	52.9	33	64.7	35	83.3	0.001

### Factors related to symptom burden, practical problems in the last week and dissatisfaction with care in the last 3 months before death

The results of the bivariate analyses are shown in Supplemental Table 2. No statistically significant interactions were found between the causes of death and any significant variables in the bivariate analyses.

Table 4 summarises the results of the final regression model of factors related to symptom burden, practical problems in the last week and dissatisfaction with care in the last 3 months. After adjusting for participant characteristics and circumstances surrounding death, individuals who died of cardiovascular disease and dementia had lower symptom burden (*p* = 0.007 and 0.001, respectively) and lower dissatisfaction (*p* = 0.04 and 0.02, respectively) than those who died of cancer. Additionally, the absence of a reliable key health professional was consistently associated with higher symptom burden and practical problems and dissatisfaction with care. Moreover, a female relative, a relative who was the patient’s child and higher practical problems were associated with dissatisfaction (*p* = 0.01, 0.008 and 0.001, respectively). These results were similar for different processing methods for missing data (Supplemental Table 3).

**Table 4.** Factors related to symptom burden and practical problems in last week and dissatisfaction with care in the last 3 months.

	Symptom burden *n* = 406			Practical problems *n* = 314			Dissatisfaction *n* = 404			*p* Value
	β	SE	*p* Value	β	SE	*p* Value	Odds ratio	95% lower	95% upper	
Cause of death										
Cancer	Ref	–	–	Ref	–	–	Ref	–	–	–
Cardiovascular disease	–2.57	0.94	0.007	–1.07	0.57	0.06	0.54	0.29	0.98	0.04
Respiratory disease	–2.35	1.31	0.07	–1.29	0.68	0.06	0.61	0.27	1.37	0.23
Dementia	–7.68	1.99	0.001	–0.53	1.20	0.66	0.14	0.03	0.71	0.02
Neurological disease	–3.10	1.94	0.11	0.45	1.02	0.66	0.42	0.13	1.36	0.15
Presence of a key health professional										
Yes	Ref	–		Ref	–		Ref	–		–
No	2.36	0.76	0.002	2.57	0.41	0.001	2.49	1.54	4.04	0.001
Confusion in the last week	2.61	0.30	0.001	0.96	0.16	0.001				
Discussion of death between professional and patient										
Yes				Ref	–					
No				1.24	0.44	0.005				
Relative’s gender										
Man							Ref	–	–	–
Woman							1.81	1.12	2.92	0.01
Relative’s relationship with the patient										
Spouse/partner							Ref	–	–	–
Son/daughter							2.24	1.23	4.07	0.008
Other							1.84	0.78	4.30	0.16
Practical problems^a^	NA			NA			1.16	1.09	1.24	0.001
	*R*^2^ = 0.20, Adj-*R*^2^ = 0.18			*R*^2^ = 0.28, Adj-*R*^2^ = 0.26			*R*^2^ = 0.17, Max-rescaled *R*^2^ = 0.23			
							Hosmer–Lemeshow test *P* = 0.78			

Blank cells indicate that these variables were not included in the final model.

a Only for dissatisfaction.

## Discussion

### Main findings

First, service use and circumstances surrounding death differed significantly across causes of death. Second, after adjusting for participant characteristics and circumstances surrounding death, symptom burden and dissatisfaction with care were significantly lower in individuals who died of cardiovascular disease or dementia than in those who died of cancer. Third, after adjustment, the absence of a reliable key health professional was associated with higher symptom burden, practical problems and dissatisfaction with care.

### What this study adds?

Differences in service use and circumstances surrounding death indicated different trajectories towards death according to cause. Although specialised palliative care is common among patients with advanced cancer, its provision for non-cancer conditions is highly variable.^31,32^ This trend is similar to that of the US data, although specialised palliative care use is more common in the US than in the UK.^13^ Additionally, general practitioners, hospital units, district/community nurses and ambulance services were common end-of-life providers, except among patients with dementia. Therefore, the provision of palliative care by these general service providers is important to enhance the quality of care for all patients at the end-of-life. Particularly, the involvement of general practitioners in generalist palliative care is crucial, considering that the majority of our sample consulted with a general practitioner in the last 3 months of life.^33^

Bereaved relatives reported that ⩾30% of patients across causes of death experienced symptoms with a score ⩾2, which indicates a moderate to overwhelming problem. However, the relief of physical and psychological symptoms remained inadequate for patients with cancer and other diseases. Additionally, 28%–38% of bereaved relatives reported dissatisfaction with the care services offered. This level seems high and similar to that in the US,^13^ where the satisfaction levels of individuals with various diseases do not significantly differ. After adjustment, individuals who died of cardiovascular disease and dementia had lower symptom burden and lower dissatisfaction than those who died of cancer, which may be because cardiovascular disease and dementia are more common among older patients. This may reflect the older age of patients and fewer symptoms in these diseases compared with the aggressive disease of cancer.

Furthermore, the absence of a reliable key health professional was associated with higher symptom burden, practical problems and dissatisfaction with care. Additionally, the effects of key health professionals did not differ by death cause or type of professional (data not shown). Importantly, this finding was consistent across causes of death. Therefore, key healthcare professionals may contribute to better care coordination and timely referrals to appropriate services for all patients.^18,21^ This implies that finding a reliable key healthcare professional and coordinated care is important at the end-of-life.

### Strength and limitations of the study

The strength of this study is the pooling of data from three studies with a large sample to obtain comparative and disease-specific results on palliative care for cancer and non-cancer diseases. The data were collected over 10 years ago. However, this was a novel data set that used validated measures to capture outcome and service data at the individual level, with pooled data enabling examination by respective cause of death from a diverse sample. The findings inform priority areas for policy of addressing inequities in palliative care provision, notably the importance of key health professionals to reduce symptom burden and concerns across diseases.

However, some limitations cannot be ignored. First, the distribution of service use and other variables may not be representative of the national population owing to the different sampling frameworks employed. Particularly, specialised palliative care use and home deaths may have been overestimated, although they were relatively low, except for cancer. Additionally, the surveys were conducted in London and the Southeast region; therefore, extrapolating the findings to national data for the UK and other countries requires care. Second, although we used pooled datasets from three mortality follow-back surveys, the sample sizes for dementia and neurological diseases were small, and the statistical power of the analysis for these diseases would have been low. Third, recall bias and unreliability due to proxy reporting are inherent limitations of the mortality follow-back survey^34,35^; family members might have overestimated symptom burden and underestimated prognosis awareness.^34,35^ Moreover, some symptoms such as nausea might be unreliable for people with dementia. Fourth, the causes of death identified by the registry might not reflect the true causes of death for older non-cancer patients, owing to the complexity and multimorbidity often observed in this population. Furthermore, the IARE1 study used cause of death data from hospital records, while others used the Office for National Statistics registry, potentially resulting in information bias. Fifth, we asked whether there were any aspects of care bereaved relatives felt unhappy about in the last 3 months of the patient’s life. This presented challenges in specifically identifying the services that bereaved relatives expressed dissatisfaction with. Sixth, the statistical power of statistical interaction is generally low and statistical model selection might produce biased results. However, bivariate analysis and sensitivity analysis support our analysis.

## Conclusions

Differences in service use and circumstances surrounding death indicate distinct trajectories towards death, depending on the cause. Improving symptom burden and satisfaction in patients and their relatives is challenging, and the presence of a reliable key health professional may be helpful for all, regardless of diagnosis.

## Supplemental Material

sj-pdf-1-pmj-10.1177_02692163241246049 – Supplemental material for Symptom burden, service use and care dissatisfaction among older adults with cancer, cardiovascular disease, respiratory disease, dementia and neurological disease during the last 3 months before death: A pooled analysis of mortality follow-back surveys Supplemental material, sj-pdf-1-pmj-10.1177_02692163241246049 for Symptom burden, service use and care dissatisfaction among older adults with cancer, cardiovascular disease, respiratory disease, dementia and neurological disease during the last 3 months before death: A pooled analysis of mortality follow-back surveys by Mitsunori Miyashita, Catherine J Evans, Deokee Yi, Barbara Gomes and Wei Gao in Palliative Medicine
