# Supplementary material for: Symptom burden, service use and care dissatisfaction among older adults with cancer, cardiovascular disease, respiratory disease, dementia and neurological disease during the last 3 months before death: A pooled analysis of mortality follow-back surveys
Source: Palliat Med. 2024 Apr 28;38(5):582–92. doi: 10.1177/02692163241246049 (PMC11107133; doi:10.1177/02692163241246049)
Supplement: sj-pdf-1-pmj-10.1177_02692163241246049 – Supplemental material for Symptom burden, service use and care dissatisfaction among older adults with cancer, cardiovascular disease, respiratory disease, dementia and neurological disease during the last 3 months before death: A pooled analysis of mortality [file sj-pdf-1-pmj-10.1177_02692163241246049.pdf]

Supplementary Table 1. Details of three original studies.

	QUALYCARE study	OPTCare Elderly study	IARE 1 study
Study participants	Bereaved relatives of people aged ≥ 18 years who died from cancer over a 1-year period	Bereaved relatives or care home staff of people aged ≥ 75 years who died from non-malignant illness or cancer in a six-month period	Bereaved carers of patients aged 65 years who died from non-malignant illness or cancer and who had accessed hospital palliative care team
Settings	Four London Primary Care Trusts: Bromley, Islington, Sutton and Merton or Westminster	Two contrasting geographical areas in southern England (rural vs city) with a total population of more than 1 million and a geographical area of 800 square miles	King's College Hospital and Guy's and St Thomas' Hospital in London
Inclusion criteria	● Deceased aged ≥ 18<br>● Deceased last resident in one of the four following Primary Care Trusts as recorded in the death registration: Bromley, Islington, Sutton and Merton or Westminster<br>● Date of death registration within 4 to 10 months before sampling	● Deceased aged ≥ 18.<br>● In the 4-10 months prior to the invitation letter and survey is to be sent out<br>● From cancer or selected non-malignant illness that common in advanced age<br>● Died in a community setting	● Deceased aged ≥ 65<br>● Accessed specialist palliative care prior to death<br>● Main informal carer aged ≥ 18 years and known to the palliative care team as the primary informal carer that was most involved in providing information

	● Cancer (ICD10 codes C00–D48) recorded as 'underlying cause of death' or in the lowest completed cause of death line in death certificate	(e.g., at home, care home or hospice) or hospital	and unpaid care for the patient<br>● Date of death registration within 4 to 10 months before sampling
Exclusion criteria	● Place of death other than an NHS acute hospital, the deceased's own home, hospice, or nursing home.<br>● Place of death unknown<br>● Deaths registered by a coroner<br>● Chosen as they provided contrasting cancer home death rates and contrasting deprivation levels within London. The study sample was then stratified by Primary Care Trust and place of death so that in each area the sample included all deaths that occurred at home, all hospice deaths, all nursing home deaths, and a random sample of NHS acute hospital deaths	● Included in the National VOICES survey administered by ONS in 2012<br>● Deaths where an informant's address is missing<br>● Deaths where an informant was an official (e.g., solicitor) or other identified person who would not be able to provide the required information	● Adults who did not provide informal (unpaid) care of an eligible patient<br>● Carers aged < 18 years<br>● Adults unable to give informed consent or deemed too ill to complete any part of the interview
Number of participants	Cancer = 328, Circulatory = 0, Respiratory = 0, Dementia = 0, Nervous system = 0; Total	Cancer = 104, Circulatory = 140, Respiratory = 88, Dementia = 51,	Cancer = 84, Circulatory = 32, Respiratory = 16, Dementia = 0,

analysed	= 328	Nervous system = 23; Total = 406	Nervous system = 19; Total = 151
Additional information	The four Primary Care Trusts were specifically chosen as they provided contrasting cancer home death rates and contrasting deprivation levels within London. The study sample was then stratified by Primary Care Trust and place of death so that in each area the sample included all deaths that occurred at home, all hospice deaths, all nursing home deaths, and a random sample of NHS acute hospital deaths.	The survey involves a random sample of bereaved relatives or carers (n = 900) of individuals aged ≥ 75 years who died in the study site. The survey is administered by the Office for National Statistics (ONS). Research team member Glickman is the Head of Analysis for the ONS and oversees the survey. The ONS used death registration data in complete confidence to identify the sampling frame. The study included two contrasting geographical areas in southern England (rural vs city) with a total population of more than 1 million and a geographical area of 800 square miles.	Participating hospitals have well-developed specialist palliative care services and are can refer dying patients to local community hospices (with inpatient beds, day care and community palliative care programs). The two palliative care teams are funded and managed by the corresponding National Health Service.

Supplementary Table 2. Results of bivariate analyses of related factors to symptom burden and practical problems at last week and dissatisfaction of care in the last 3 months.

The first P values are by ANOVA/Fisher's exact test, second are by trend test (Pearson's correlation coefficients or Mantel test).

	Symptom burdens				Practical problems				Dissatisfaction				
									Dissatisfied		Satisfied		
	n	Mean	SD	P-value	n	Mean	SD	P-value	n	%	n	%	
Patient's characteristics													
Cause of death													
Cancer (C00-C97)	304	19.3	7.7	0.082	304	8.3	3.9	0.008	196	39.4	302	60.6	0.224
Cardiovascular disease(I00-I99)	84	17.1	7.8	0.073	84	8.7	3.8		51	31.3	112	68.7	
Respiratory diseases (J00-J99)	44	18.7	7.6		44	8.8	3.9		33	34.7	62	65.3	
Dementia (F00-F03)	19	16.3	5.8		19	10.1	2.7		14	28.6	35	71.4	
Neurological diseases (G00-G99)	16	20.7	9.6		16	11.6	4.0		17	42.5	23	57.5	
Patient's age													
75-79	90	20.5	7.5	0.071	90	8.6	4.2	0.977	67	40.6	98	59.4	0.632
80-84	154	17.9	7.6	0.347	154	8.5	3.8	0.070	94	37.9	154	62.1	0.11

													1
85-89	109	19.4	7.5		109	8.5	3.8		78	36.3	137	63.7	
90-94	86	18.1	8.0		86	8.8	3.9		55	34.0	107	66.1	
95-	28	17.6	8.5		28	8.7	4.1		17	30.9	38	69.1	
Patient's gender													
Male	208	18.5	8.0		208	8.5	3.9	0.684	134	35.6	242	64.4	0.52
				0.498									9
Female	259	19.0	7.5		259	8.7	3.9		177	37.7	292	62.3	
Patient's marital status													
Married/with partner	154	18.3	7.8		154	9.1	3.9	0.202	88	33.9	172	66.2	0.31
				0.597									6
Widowed	240	19.2	7.5		240	8.5	3.8		164	39.7	249	60.3	
Divorced /separated/never married	33	17.9	6.9		33	7.6	3.9		34	37.0	58	63.0	
Do not know/missing	40	18.4	9.4		40	8.5	4.3		25	31.3	55	68.8	
Patient's religion													
No religion	57	18.2	8.1		57	7.6	4.1	0.099	40	41.2	57	58.8	0.29
				0.835									3
Christian	380	18.8	7.6		380	8.8	3.9		255	36.5	444	63.5	
Other	19	18.9	8.6		19	8.3	3.4		8	25.8	23	74.2	
Missing	11	20.5	8.7		11	8.0	4.1		8	44.4	10	55.6	0.40
													5
Patient's ethnic group													
White British	417	18.6	7.7	0.226	417	8.6	3.9	0.603	263	35.3	483	64.8	0.01

													5
Other	35	20.2	8.2		35	9.0	4.3		38	49.4	39	50.7	
Missing	15	20.5	8.2		15	7.6	3.0						
Patient's living circumstances													
Living alone	221	18.1	7.0	0.190	221	8.0	3.8	0.017	139	36.8	239	63.2	0.13
													9
With respondent only	120	19.0	8.0		120	9.2	4.1		71	32.1	150	67.9	
With respondent and others	48	20.9	8.9		48	9.1	4.0		39	45.9	46	54.1	
With other people only	43	18.5	8.9		43	8.9	3.7		31	44.9	38	55.1	
Care home	30	20.1	8.3		30	9.7	3.9		29	35.4	53	64.6	
Missing	5	16.6	4.8		5	10.0	2.2		2	20.0	8	80.0	
Financial status													
Living comfortably	207	18.7	8.0		207	8.2	4.0	0.168	139	36.8	239	63.2	0.60
				0.777									2
Doing alright	165	18.5	7.5		165	8.8	3.9		107	37.2	181	62.9	0.63
								0.029					2
Just about getting by	50	19.0	7.1		50	9.2	3.5		29	32.6	60	67.4	
Finding it quite difficult/finding it very difficult	34	20.0	8.5		34	9.4	3.9		29	43.9	37	56.1	
Missing	11	18.6	8.1		11	8.6	3.5		7	29.2	17	70.8	
Length of illness													
Less than a week	12	11.8	8.6		12	6.9	3.3	0.174	7	21.2	26	78.8	0.06
				0.022									2

Less than one month	39	18.7	7.3		39	9.4	2.8		39	46.4	45	53.6	0.77
				0.004				0.562					1
Less than six months	129	18.6	6.9		129	8.6	3.8		78	36.8	134	63.2	
Less than one year	72	19.3	8.4		72	8.6	4.0		46	38.7	73	61.3	
Less than three years	113	19.9	7.4		113	9.1	4.2		73	39.0	114	61.0	
Three years or more	90	18.9	7.7		90	8.1	3.7		64	34.4	122	65.6	
Missing	12	13.7	11.3		12	7.3	5.1		4	16.7	20	83.3	
Patient's awareness of prognosis													
Yes	319	18.7	7.4		319	8.1	3.8	0.000	195	37.0	332	63.0	0.87
				0.929									8
No	148	18.8	8.4		148	9.8	4.0		116	36.5	202	63.5	
Confusion in last week													
No, not at all	127	14.7	7.4		127	7.2	4.0	0.000	78	35.9	139	64.1	0.80
				0.000									7
Occasionally	138	18.1	7.4		138	7.6	3.5		69	33.3	138	66.7	0.45
				0.000				0.000					2
Sometimes	93	21.3	6.6		93	9.0	3.4		64	39.8	97	60.3	
Most of the time	70	22.5	6.8		70	10.5	3.2		59	39.3	91	60.7	
Always	38	22.0	7.4		38	12.5	3.5		34	36.6	59	63.4	
Missing	1	19.0	.		1	9.0	.		7	41.2	10	58.8	
Unconscious/coma in last week													
No, not at all	258	18.3	7.8		258	8.4	4.0	0.000	159	36.0	283	64.0	0.36
				0.190									1

Occasionally	79	18.8	7.3		79	7.6	3.5		44	36.1	78	63.9	0.86
				0.171				0.000					6
Sometimes	83	20.3	7.5		83	9.5	3.6		58	42.3	79	57.7	
Most of the time	40	19.7	7.9		40	10.3	3.7		36	34.0	70	66.0	
Always	5	14.8	10.9		5	11.4	2.9		4	22.2	14	77.8	
Missing	2	12.0	7.1		2	4.0	0.0		10	50.0	10	50.0	
Place of death													
Home	162	17.4	8.1		162	8.0	4.0	0.002	93	40.1	139	59.9	0.07
				0.012									5
Hospice	77	18.6	7.3		77	7.8	3.9		43	30.9	96	69.1	
Hospital	152	20.2	7.9		152	9.2	3.7		127	39.9	191	60.1	
Care home	76	19.0	6.3		76	9.5	3.8		48	30.8	108	69.2	
Bereaved relative's characteristics													
Relative's gender													
Male	156	17.8	6.9		156	8.2	3.7	0.120	82	29.8	193	70.2	0.01
				0.059									3
Female	310	19.3	8.1		310	8.8	4.0		228	40.1	340	59.9	
Missing	1	6.0	.		1	5.0	.		1	50.0	1	50.0	
Relative's age													
-44	20	17.9	9.5		20	6.1	3.9	0.008	13	38.2	21	61.8	0.00
				0.097									0
45-54	119	19.8	7.2	0.000	119	9.4	3.6	0.020	100	48.8	105	51.2	0.00

													0
55-64	155	19.5	7.6		155	8.6	3.9		107	38.6	170	61.4	
65-74	93	17.8	8.4		93	8.7	3.8		58	31.9	124	68.1	
75-84	65	17.0	6.9		65	8.2	4.3		24	20.5	93	79.5	
85-	12	17.7	7.1		12	7.3	3.2		8	30.8	18	69.2	
Missing	3	14.3	11.9		3	5.7	2.1		1	25.0	3	75.0	
Relative's relationship to patient													
Spouse/partner	99	18.0	7.8		99	8.5	4.1	0.980	45	24.9	136	75.1	0.00
				0.513									1
Son/daughter	307	19.0	7.9		307	8.6	3.9		219	40.9	316	59.1	
Other	61	19.1	6.9		61	8.6	3.8		47	36.4	82	63.6	
Relative's religion													
No religion	71	18.7	7.0		71	7.9	4.0	0.232	63	42.9	84	57.1	0.17
				0.789									4
Christian	359	18.8	7.8		359	8.8	3.9		225	35.3	412	64.7	
Other	28	19.8	8.5		28	8.9	3.3		19	43.2	25	56.8	
Missing	9	16.6	9.3		9	6.3	3.7		4	23.5	13	76.5	
Relative's ethnic group													
White British	428	18.6	7.7		428	8.6	3.9	0.158	272	35.4	496	64.6	0.00
				0.127									2
Other	26	21.0	8.7		26	9.7	4.1		32	56.1	25	43.9	
Missing	13	19.5	8.0		13	7.5	2.9		7	35.0	13	65.0	
Relative's awareness of prognosis													

Yes	367	19.1	7.5	0.099	367	8.4	3.9	0.036	228	35.4	417	64.7	0.115
No	100	17.6	8.5		100	9.3	4.0		83	41.5	117	58.5	
Relative's presence at time of death													
Yes	285	18.5	7.6	0.399	285	8.3	3.9	0.016	187	38.8	295	61.2	0.167
No/do not know/missing	182	19.1	7.9		182	9.1	3.9		124	34.2	239	65.8	
Service Uses													
ICU stay													
Yes	48	19.6	6.9	0.510	48	8.8	3.7	0.560	37	38.5	59	61.5	0.932
No	384	18.6	7.8		384	8.5	4.0		251	36.6	435	63.4	
Do not know/ missing	35	19.7	8.0		35	9.2	3.1		23	36.5	40	63.5	
Hospital stay													
Yes	303	19.5	7.9	0.011	303	8.9	3.9	0.054	231	41.8	322	58.2	0.000
No	159	17.4	7.3		159	8.0	3.9		76	26.9	207	73.1	
Do not know/ missing	5	15.2	4.4		5	9.0	2.1		4	44.4	5	55.6	
Hospice stay													
Yes	83	18.7	7.4	0.539	83	7.7	3.9	0.084	48	30.4	110	69.6	0.108
No	379	18.8	7.7		379	8.8	3.9		261	38.5	417	61.5	

Do not know/ missing	5	15.0	12.8		5	8.4	3.4		2	22.2	7	77.8	
Nursing home stay													
Yes	85	19.3	6.6	0.347	85	9.4	4.0	0.110	60	34.9	112	65.1	0.84
													2
No	374	18.7	8.0		374	8.4	3.9		245	37.3	412	62.7	
Do not know/ missing	8	15.3	4.4		8	8.0	3.3		6	37.5	10	62.5	
Residential home stay													
Yes	32	18.8	7.3		32	9.1	3.9	0.771	24	32.0	51	68.0	0.62
				0.277									8
No	425	18.9	7.8		425	8.6	3.9		280	37.2	473	62.8	
Do not know/ missing	10	14.9	3.8		10	8.3	3.0		7	41.2	10	58.8	
Outpatient clinic visit													
Yes	114	18.1	7.5		114	8.2	4.0	0.344	74	37.2	125	62.8	0.98
				0.424									3
No	334	19.1	7.8		334	8.8	3.9		222	36.8	382	63.3	
Do not know/ missing	19	17.8	8.1		19	8.2	3.6		15	35.7	27	64.3	
Day care visit													
Yes	24	21.1	7.9		24	8.5	4.3	0.932	16	34.8	30	65.2	0.94
				0.164									5
No	420	18.7	7.7		420	8.6	3.9		280	37.0	477	63.0	
Do not know/ missing	23	16.8	7.0		23	8.9	3.8		15	35.7	27	64.3	
Ambulance services use													
Yes	278	19.2	7.7	0.324	278	8.7	4.0	0.647	191	37.5	318	62.5	0.66

													6
No	174	18.1	7.9		174	8.4	3.9		109	35.2	201	64.8	
Do not know/ missing	15	19.3	5.7		15	9.3	2.9		11	42.3	15	57.7	
GP contact face-to-face													
Yes	426	18.9	7.7		426	8.7	3.9	0.572	284	37.7	469	62.3	0.28
				0.603									1
No	39	17.6	8.2		39	8.0	3.6		24	28.9	59	71.1	
Do not know/ missing	2	17.5	7.8		2	9.5	6.4		3	33.3	6	66.7	
GP contact by telephone													
Yes	267	18.9	7.6	0.709	267	8.5	3.8	0.681	188	42.3	256	57.7	0.00
													2
No	185	18.7	7.8		185	8.8	4.1		110	30.4	252	69.6	
Do not know/ missing	15	17.2	9.8		15	8.1	3.6		13	33.3	26	66.7	
District nurse contact													
Yes	300	19.3	7.7	0.154	300	8.6	4.0	0.659	184	36.4	321	63.6	0.62
													5
No	160	17.8	7.8		160	8.7	3.7		118	36.8	203	63.2	
Do not know/ missing	7	19.3	6.3		7	7.3	2.6		9	47.4	10	52.6	
Marie Curie contact													
Yes	59	19.3	7.5		59	8.1	3.9	0.466	31	36.5	54	63.5	0.52
				0.846									2
No	383	18.7	7.9		383	8.7	3.9		265	37.4	444	62.6	
Do not know/ missing	25	19.0	6.1		25	8.2	3.3		15	29.4	36	70.6	

Macmillan or other specialist nurse contact													
Yes	144	19.3	7.2		144	8.2	3.4	0.191	73	32.4	152	67.6	0.28
				0.271									6
No	310	18.4	8.0		310	8.8	4.1		223	38.4	358	61.6	
Do not know/ missing	13	21.2	6.7		13	8.0	3.3		15	38.5	24	61.5	
Other nurse contact													
Yes	59	17.7	6.8		59	8.7	4.3	0.967	57	41.9	79	58.1	0.23
				0.329									3
No	326	18.7	7.9		326	8.6	3.9		197	34.9	368	65.1	
Do not know/ missing	82	19.7	7.5		82	8.6	3.8		57	39.6	87	60.4	
Palliative care team contact													
Yes	174	19.3	7.6		174	8.2	3.9	0.089	111	41.6	156	58.4	0.11
				0.383									2
No	275	18.4	7.8		275	8.9	3.9		189	35.0	351	65.0	
Do not know/ missing	18	19.7	7.6		18	7.9	3.4		11	29.0	27	71.1	
Physiotherapist contact													
Yes	53	20.9	7.7		53	8.4	3.9	0.611	42	43.8	54	56.3	0.32
				0.044									5
No	398	18.4	7.7		398	8.7	3.9		257	35.9	459	64.1	
Do not know/ missing	16	20.9	6.8		16	7.8	3.8		12	36.4	21	63.6	
Occupational therapist contact													
Yes	85	20.4	7.9		85	8.1	4.0	0.420	58	43.6	75	56.4	0.09
				0.048									9

No	369	18.3	7.6		369	8.7	3.8		240	35.1	444	64.9	
Do not know/ missing	13	21.2	8.1		13	8.5	4.4		13	46.4	15	53.6	
Psychiatrist contact													
Yes	13	19.2	5.6		13	8.8	4.2	0.356	9	34.6	17	65.4	0.97
				0.889									3
No	440	18.7	7.8		440	8.6	3.9		291	36.9	498	63.1	
Do not know/ missing	14	19.6	7.2		14	7.1	4.1		11	36.7	19	63.3	
Psychologist or counsellor contact													
Yes	14	22.9	8.0		14	9.9	3.5	0.309	8	44.4	10	55.6	0.68
				0.093									7
No	439	18.6	7.7		439	8.6	3.9		291	36.5	507	63.5	
Do not know/ missing	14	20.4	7.8		14	7.7	3.6		12	41.4	17	58.6	
Spiritual care contact													
Yes	93	18.7	7.8		93	8.3	4.0	0.554	67	43.8	86	56.2	0.07
				0.816									8
No	364	18.8	7.7		364	8.7	3.9		233	34.9	435	65.1	
Do not know/ missing	10	20.3	8.2		10	7.8	3.9		11	45.8	13	54.2	
Social worker contact													
Yes	77	20.5	7.6		77	9.7	4.3	0.017	55	38.5	88	61.5	0.87
				0.094									2
No	375	18.4	7.7		375	8.4	3.8		244	36.4	427	63.6	
Do not know/ missing	15	18.6	7.1		15	7.7	3.4		12	38.7	19	61.3	
Other professionals contact													

Yes	49	19.7	8.5	0.265	49	9.1	4.0	0.595	36	45.6	43	54.4	0.117
No	365	18.5	7.7		365	8.5	3.9		233	35.1	431	64.9	
Do not know/ missing	53	20.0	7.2		53	8.6	3.7		42	41.2	60	58.8	
Any specialist palliative care contact													
No	128	17.3	7.7	0.009	128	9.3	3.9	0.020	87	32.0	185	68.0	0.045
Yes	339	19.3	7.7		339	8.3	3.9		224	39.1	349	60.9	
Presence of key health professional													
Yes	263	17.8	7.8	0.002	263	7.5	3.6	0.000	132	29.3	319	70.7	0.000
No	149	20.6	7.6		149	10.3	3.7		152	54.5	127	45.5	
Do not know/ missing	55	18.2	6.9		55	9.0	4.1		27	23.5	88	76.5	
Discussion of death between professional and patient													
Yes	210	18.7	7.6	0.974	210	7.5	3.6	0.000	112	36.1	198	63.9	0.807
No	162	18.8	7.6		162	9.5	3.9		114	36.2	201	63.8	
Do not know/ missing	95	18.9	8.4		95	9.5	4.0		85	38.6	135	61.4	
Discussion of death between professional and relative													
Yes	326	19.3	7.7	0.099	326	8.4	3.8	0.217	205	35.1	379	64.9	0.255
No	125	17.7	7.4		125	9.1	4.1		91	39.9	137	60.1	
Do not know/ missing	16	17.0	9.6		16	9.1	4.4		15	45.5	18	54.6	

Supplementary Table 3. Sensitivity analyses for related factors to symptom burden, practical problems at last week and dissatisfaction of care in the last 3 months.

Including categories of "do not know/missing"

	Symptom burdens n=461			Practical problems n=437			Dissatisfaction n=458			
	ß	SE	P-value	ß	SE	P-value	Odds Ratio	95% lower	95% upper	P-value
Cause of death										
Cancer	ref	-	-	ref	-	-	ref	-	-	-
CVD*	-2.48	0.88	0.005	-0.30	0.47	0.5303	0.53	0.30	0.95	0.0324
Respiratory disease	-2.11	1.16	0.0698	-1.13	0.58	0.0527	0.63	0.30	1.31	0.214
Dementia**	-3.02	1.85	0.1033	0.43	0.93	0.6409	0.38	0.12	1.20	0.0978
Neurological disease	-7.70	1.78	< .0001	-1.11	0.90	0.2192	0.21	0.05	0.82	0.0244
Presence of key health professional										
Yes	ref	-					ref	-	-	-
No	2.35	0.74	0.0017	2.55	0.37	< .0001	2.47	1.53	3.99	0.0002
Do not know/ missing	0.71	1.05	0.4967	1.22	0.53	0.0217	0.64	0.31	1.32	0.2312
Confusion in last week	2.57	0.28	< .0001	1.04	0.14	< .0001				
Discussion of death between professional and patient										
Yes										

No				1.20	0.41	0.0037				
Do not know/ missing				1.55	0.45	0.0006				
Relative's gender										
Male							ref	-	-	-
Female							1.76	1.12	2.78	0.0147
Relative's relationship to patient										
Spouse/partner							ref	-	-	-
Son/daughter							2.07	1.19	3.60	0.0104
Other							1.83	0.84	3.98	0.1295
Practical problems (*)							1.17	1.10	1.24	< .0001
	R2 = 0.20, Adj-R2 = 0.18			R2 = 0.27, Adj-R2 = 0.26			R square = 0.17, Max-rescaled R2 = 0.23 Hosmer-Lemeshow test P = 0.70			

Blank cells indicate these variables were not included in the final model.
*only for dissatisfaction.

Using available all data by imputing means values for symptoms and practical problems

	Symptom burdens n = 807			Practical problems n = 848			Dissatisfaction n = 841			
	ß	SE	P-value	ß	SE	P-value	Odds Ratio	95% lower	95% upper	P-value
Cause of death										
Cancer	ref	-	-	ref	-	-	ref	-	-	-
Circulatory	-2.06	0.78	0.0086	-0.12	0.37	0.7533	0.53	0.35	0.80	0.0028

Respiratory	-1.22	0.94	0.1963	-0.60	0.42	0.1549	0.72	0.43	1.20	0.2082
Nervous system	-2.47	1.43	0.085	0.56	0.66	0.3942	0.65	0.32	1.32	0.2315
Dementia	-4.79	1.38	0.0006	0.22	0.63	0.7233	0.37	0.19	0.75	0.0058
Presence of key health professional										
Yes	ref	-					ref	-	-	-
No	2.21	0.66	0.0009	2.06	0.30	< .0001	2.26	1.61	3.16	<.0001
Do not know/ missing	1.89	0.88	0.0322	1.75	0.40	< .0001	0.59	0.35	0.99	0.0452
Confusion in last week	2.35	0.24	<.0001	1.15	0.11	< .0001				
Discussion of death between professional and patient										
Yes										
No				1.28	0.34	0.0002				
Do not know/ missing				1.50	0.36	< .0001				
Bereaved relative's gender										
Male							ref	-	-	-
Female							1.59	1.14	2.22	0.0068
Bereaved relative's relationship to patient										
Spouse/partner							ref	-	-	-
Son/daughter							2.18	1.44	3.29	0.0002
Other							1.82	1.06	3.11	0.0292
Practical problems (*)							1.14	1.10	1.19	< .0001
	R2 = 0.20, Adj-R2 = 0.18			R2 = 0.12, Adj-R2 = 0.12			R square = 0.14, Max-rescaled R2 = 0.20			

Hosmer-Lemeshow test P = 0.33

Blank cells indicate these variables were not included in the final model.

*only for dissatisfaction.
